# Using the Countermovement Jump Metrics to Assess Dynamic Eccentric Strength: A Preliminary Study

**DOI:** 10.3390/ijerph192316176

**Published:** 2022-12-03

**Authors:** Chien-Chun Chang, Chieh-Ying Chiang

**Affiliations:** 1 Graduate Institute of Athletics and Coaching Science, National Taiwan Sport University, Taoyuan 333, Taiwan; 2 Department of Sports Training Science-Combats, National Taiwan Sport University, Taoyuan 333, Taiwan

**Keywords:** biomechanics, kinetics, strength test

## Abstract

Background: This study aimed to determine the validity and reliability of the countermovement jump (CMJ) as a dynamic eccentric (Ecc) strength test. Methods: Thirty-three college male student-athletes were recruited to participate in this study. The participants first performed CMJs with the second consisting of one repetition maximum back squat (1RM-BS) test. CMJ and 1RM-BS tests were performed on twin force plates. Results: The CMJ had significant correlations with the Ecc peak force (EccPF), and Ecc mean force (EccMF) of 1RM-BS, respectively (*r* = 0.61–0.69). Moreover, all parameters had a coefficient of variation (CV) < 10%. The intraclass correlation coefficient (ICC) values were moderate to excellent for each metric using the CMJ (0.94–0.97). The 1RM-BS and CMJ EccPF, EccMF Bland-Altman bias estimate variance ratio is 1.31–1.67, showing a moderate-large correlation in the Bland-Altman plot. Conclusions: CMJ ECC phase kinetics were associated with the 1RM-BS EccPF and EccMF. The CMJ can be an alternative tool for eccentric dynamic strength assessment.

## 1. Introduction

Strength assessment is crucial in understanding athletic performance. It is a measure of maximum force capability or production [1], and it also underpins many aspects of sports performance [2]. Dynamic strength tests using free weight exercises are the most commonly used in strength assessment due to their accessibility. Thus, one repetition maximum (1RM) load being lifted refers to athletes’ maximum dynamic strength [3]. The 1RM back squat (1RM-BS) assessment is a well-established, valid, and reliable method of determining maximal strength. The 1RM-BS measures the maximum amount of load lifted with proper form [1] and has consistently been shown to be a reliable test in a range of populations and exercises [4,5,6,7,8]. Muscle contractions can be divided into eccentric (ECC), isometric (ISO), and concentric (CON) contractions [9]. Ecc contractions produce greater levels of force than Iso and Con contractions [10,11]. Ecc strength is a critical aspect of the ability to change direction, stop, and decelerate [12,13,14,15,16], and it is important to decelerate at the end of the range of motion [13]. Many sports require the ability to tolerate large Ecc forces [17]. The relationship between Ecc strength characteristics and dynamic sports performance has been previously explored and may explain favorable changes in strength, jumping, and sprinting ability following Ecc-based training interventions [12,18,19]. Therefore, being able to assess Ecc strength can provide useful insights into sports performance. Measuring Ecc strength can also provide information about the responses to training adaptations [20,21]. Further, the Ecc phase of 1RM-BS has been reported as a useful testing tool to measure Ecc strength; specifically, the load being lifted and the kinetic and kinematic parameters were used to present the Ecc strength characteristics [1,13,19,22].

Ecc strength is critical for performing rapid stretch-shortening cycle (SSC) activities such as the countermovement jump (CMJ) [23,24]. The SSC is the physiological mechanism of an Ecc muscular and tissue stretch phenomenon in which elastic potential energy stored during the Ecc phase is reutilized to augment the subsequent Con action [25]. One of the reasons for this improved force production is an increase in the number of cross bridges, because Ecc strength is mechanical and thus a consequence of the number of cross-bridges formed [23,26,27]. Therefore, the relevance of the Ecc strength of athletes in sports performance to the assessment of the jumping Ecc phase characteristics of athletes would appear pertinent [21,24,28]. Ecc strength is typically assessed using isokinetic dynamometry, whereby the velocity is controlled during the task and the force is measured as it increases with velocity [29,30]. However, these assessments are limited to single-joint tasks [24]. Studies have suggested that single-joint isometric tests are poor predictors of performance in multipoint explosive movements such as a CMJ [31]. Through the use of force platform kinetics analysis technologies, it may be possible to assess the Ecc phase characteristics using CMJs. Force plates are considered the gold standard because they enable the acquisition of force-time data kinetics, and kinematic parameters can then be obtained by calculation. Thus, practitioners have the ability to quantify both outcome measures [4,32]. At present, there are limited Ecc strength testing options available to practitioners. Therefore, CMJ kinetics may provide valuable insight into the characteristics of an individual’s Ecc strength. This study aimed to use the CMJ to examine its relationship with the 1RM-BS and to analyze the validity and reliability of CMJ Ecc phase parameters, including PF and MF. This may lead to the validation of CMJ as an evaluation method for Ecc strength.

## 2. Materials and Methods

This study used a single-session design modified from the methods of a previously validated study for the 1RM-BS and CMJ [19,32]. Before the formal test, the participants performed a 5–10 min dynamic warm-up. The warm-up included 25 jumping jacks, 30 high knee runs, 15 bodyweight squats, 20 side lunges, and 5 practice trials of the CMJ at their perceived maximal effort. Subjects came to the laboratory to familiarize themselves with the process and understand and agree to the experimental risks and precautions. They performed the CMJ test first, with a 1–2 min interval for each test. After the CMJ test, the 1RM-BS test was performed after a 30 min seated rest, and each subject’s squat depth and load were recorded for formal experiments to preadjust the squat depth standard of each subject. Formal experiments were conducted at 3-day intervals. After a standardized warm-up, CMJ and 1RM-BS were sequentially performed on twin force plates (9260AA, Kistler Group, Winterthur, Switzerland) for data collection.

### 2.1. Participants

Thirty-three college male student-athletes (age: 20.24 ± 1.75 years; body height: 174.93 ± 9.87 cm; body mass: 78.43 ± 12.82 kg), who had been familiarized with the experimental content and procedures before the experiment, volunteered to participate in the present study. The qualification conditions were a minimum of 2 years of strength training experience and no lower extremity injuries within 1 month before the experiment. All subjects were required to provide written informed consent.

### 2.2. Countermovement Jump Assessment

Participants performed a CMJ on dual force plates (9260AA, Kistler Group, Winterthur, Switzerland) with sampling at 1000 Hz. The participants were instructed to step onto the force plates, stand with their feet shoulder-width apart, and remain motionless for 2 s so that the system weight could be accurately determined [33,34]. The participants positioned their hands on a polyvinyl chloride (PVC) bar that was placed across their shoulders [35]. Participants were instructed to get in the “ready position”, which consisted of the athlete firmly holding the PVC pipe (0 kg) and a countdown of “3, 2, 1, jump” was given. Participants performed each jump for maximal height, with the eccentric phase depth self-selected to avoid any alterations in their preferred jump strategy, and the legs were required to remain fully extended during the flight phase of the jump [18,34,36]. Ground reaction force (GRF) data were collected using a USB data acquisition system (Type 5695B, Kistler Group, Winterthur, Switzerland). Data were analyzed using Bioware software (Version 5.0.3; Bioware Software Type 2812A, Kistler Group, Winterthur, Switzerland) with the force (recorded in newtons [N]) determined in 2 CMJs [13]. The force value of the subject standing on the force plate for 1 s was defined as the stabilization phase, and the first 30 milliseconds was the time point for any first meaningful change in force value [37,38]. The eccentric phase of the CMJ was defined as occurring between peak negative and 0 center of mass velocity. The concentric phase of the CMJ was defined as occurring between the instant at which COM velocity exceeded 0.01 m·s^−1^ and the instant of take-off [34]. The recorded parameters included jump height (JH), which was determined from the flight time (FT); the formula is 1/2 × g × (t/2)^2^, where g = 9.81 m/s^2^. FT is determined from the time point when the force value is lower than 10 N before jumping off the ground until the time point when the first strength value is greater than 10 N when the subject touches the ground [39]. The eccentric peak force (EccPF) is determined from the maximum GRF generated during the Ecc phase, and the eccentric mean force (EccMF) is determined from the average GRF generated during the Ecc phase.

### 2.3. Back Squat Testing

Dynamic strength was assessed using a 1 repetition maximum back squat (1RM-BS) on dual force plates (9260AA, Kistler Group, Winterthur, Switzerland) with sampling at 1000 Hz. Before the formal test, participants were instructed to position their feet shoulder-width apart with an unloaded Olympic bar positioned across the top of their shoulders (trapezius muscle) and the depth of the BS was determined to descend and lower to at least a half squat position (90° knee angle) [13,40]. A goniometer measured the knee angle, and light gates (Fusion Sport Smart Speed Timing Gates, Brisbane, Australia) were placed outside the squat rack as an external reference for the required squat depth.

A warm-up following the guidelines from [41] consisted of 5 repetitions at 40% of the subjects’ perceived maximal effort. After a 3 min rest period, subjects performed 5 repetitions at 80% of their perceived maximal effort. After another 3 min rest period, the weight was increased, with subjects performing 1 repetition at the new load, resting another 3 min before increasing the load, and conducting formal testing. The loaded was continually increased (4–7 kg) for each trial, and a maximum of 5 attempts at any given load was permitted until the participants could not perform the repetition; the maximal load being lifted was recorded for the analysis [42]. Ground reaction force (GRF) data were collected using a USB data acquisition system (Type 5695B, Kistler Group, Winterthur, Switzerland). Data were analyzed using Bioware software (Version 5.0.3; Bioware Software Type 2812A, Kistler Group, Winterthur, Switzerland) with the force (recorded in newtons [N]) determined during 1RM-BS. The system velocity during 1RM-BS was calculated by integrating the acceleration data with the force-time data; the force value of the subject started when standing on the force plate to stabilize for 1 s, which was defined as the stabilization phase, and the onset of movement was defined as the time point at which the force value was reduced by more than 5 standard deviations of the system weight [37,43]. The time from the onset movement to a system velocity of zero was distinguished as the eccentric and concentric phase. The point at which the acceleration became lower than zero was considered the start of the eccentric phase [43,44,45]; the eccentric peak force (EccPF) was determined from the maximum GRF generated during the Ecc phase, and the eccentric mean force (EccMF) was determined from the average GRF generated during the Ecc phase.

### 2.4. Statistical Analysis

All data reported are the means ± SDs. Data normality was determined using the Kolmogorov-Smirnov test (*p* > 0.05). Within-session reliability was computed for both CMJ and 1RM-BS measurements using the coefficient of variation (CV), and CV values less than 10% were deemed acceptable [46]. CMJ was analyzed using a two-way random intraclass correlation coefficient (ICC). The ICC values were interpreted in accordance with guidelines where >0.90 = excellent, 0.75–0.90 = good, 0.50–0.74 = moderate, and <0.50 = poor [47]. Absolute agreement and 95% confidence intervals (CIs) were used [48]. The levels of agreement between the CMJ and the 1RM-BS eccentric parameters were determined from Bland-Altman plots with 95% upper and lower limits [49]. Correlations between the measured CMJ and 1RM-BS eccentric parameters were calculated using Pearson’s product-moment (*r*). The strength of the relationship was classified as small (0.10–0.29), moderate (0.30–0.49), large (0.50–0.69), very large (0.70–0.89), and nearly perfect (0.90–1.00) [50]. The suitability of the sample size to use partial correlation analysis was based on sample size tables suggested by [51,52]. Further, G-Power was used to assess the validity of the sample size to conduct a partial correlation analysis.

## 3. Results

In this study’s G-Power test results the minimum sample size for Pearson’s r method is 23 subjects, and according to previous research a sample size equal or superior to 25 suffices [51]. The CMJ and 1RM-BS data were determined to be normally distributed (*p* > 0.05), and reliability statistics with the mean ± SD data are shown in Table 1. Both CMJ and 1RM-BS EccPF and EccMF showed acceptable CV values for each test (CMJ ≤ 3.81%; 1RM-BS ≤ 9.75%). The ICC values were moderate to excellent for each metric when using CMJ (0.94–0.97). Pearson’s r values were as follows: the CMJ was significantly correlated with EccPF and EccMF of 1RM-BS at a high level (*r* = 0.69 ** *p* = 0.01; *r* = 0.61 ** *p* = 0.01) (Table 2).

**Figure 1:**
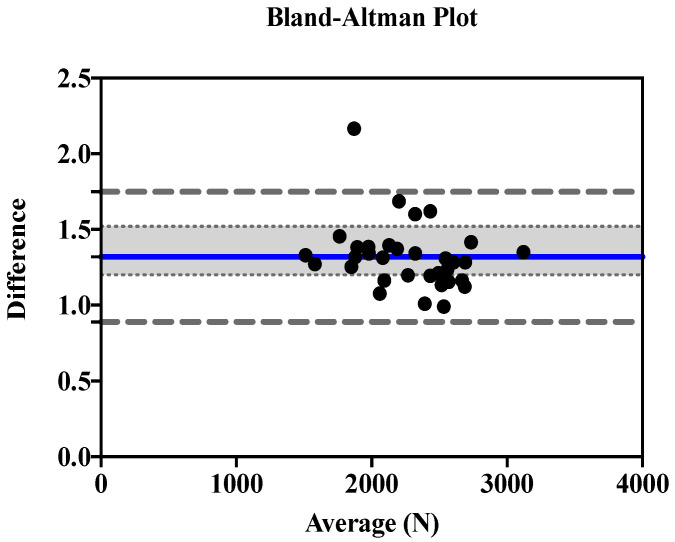
Bland-Altman plot depicting the levels of agreement for the EccPF of the 1RM-BS and CMJ, including bias estimate (1.31) and both lower (0.88) and upper (1.74) limits of agreement.

**Figure 2:**
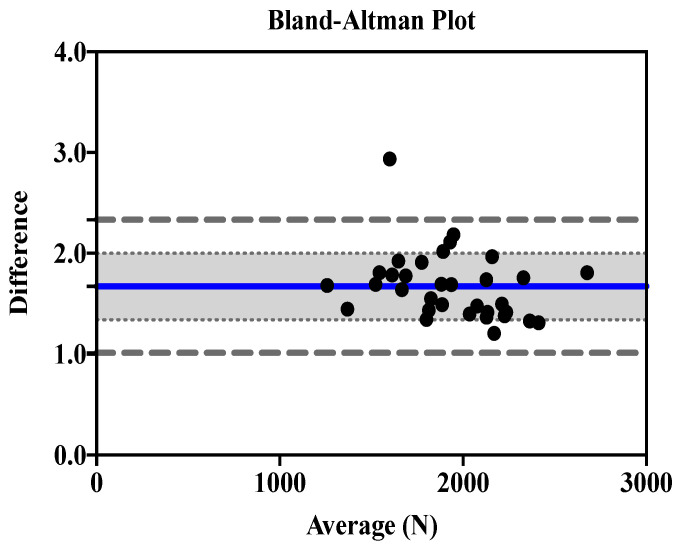
Bland-Altman plot depicting the levels of agreement for the EccMF of the 1RM-BS and CMJ, including bias estimate (1.67) and both lower (1.01) and upper (2.33) limits of agreement.

Figure 1 and Figure 2 show Bland-Altman plots of the EccPF for the 1RM-BS and CMJ (ratio bias estimate, 1.31; lower and upper limits of agreement, 0.88 and 1.74) (Figure 1), and the EccMF for the 1RM-BS and CMJ (ratio bias estimate, 1.67; lower and upper limits of agreement, 1.01 and 2.33) (Figure 2). 

## 4. Discussion

The present study aimed to establish the validity and reliability of the CMJ as a dynamic Ecc strength test. The results showed that the CMJ had large correlations with the EccPF and EccMF of the 1RM-BS, respectively (*r* = 0.61–0.69), and all CVs were <10%. The CMJ and 1RM-BS test Ecc phase parameters have acceptable reliability and validity and are significantly correlated. The Ecc strength measurement method and the correlation and consistency of the PF and MF parameters in the Ecc phase of the 1RM-BS and CMJ is worth further exploration. The findings of the current study suggest that the 1RM-BS or squat device analysis of EccPF has good reliability across loading conditions, and the EccMF has CV values under the commonly applied threshold of 10% [19,23]. Previous investigations have reported different methods of quantifying force-time variables, noting that peak values may be a more reliable measure [53]. Stock and Luera (2014) demonstrated moderate-to-high test-retest reliability for the EccPF and EccMF values obtained from maximal Con and Ecc squat testing [54]. McNeill et al. (2021) investigated the reliability of peak and mean force values during the Ecc phase of a back squat test in trained athletes (CV: 3.2%, 3.8%) [19]. There is currently research utilizing Ecc squat to determine maximal Ecc strength [12,13,55], but this is mainly considered dependent on the subjective determination of the failure threshold [19]. However, maximal Ecc strength testing typically relies on hefty loading and forces, which may limit the applicability in sports due to potential muscle damage, muscle soreness, decreased sports performance, and recovery [19,56]. This confirms that 1RM-BS can be used as a test standard for Ecc strength and establishes the feasibility of CMJ as a test method.

Previous studies have reported strong relationships between maximal squat strength and CMJ performance [57,58,59]. However, to the authors’ knowledge, this was the first study to separately investigate the relationships between the Ecc phase force value during a 1RM-BS and CMJ. As proposed in other studies, Ecc force production is mechanical and dependent on the number of active actin-myosin cross bridges [60,61]. The greater levels of Ecc strength during the squat resulted from maintaining a more significant number of cross-bridges throughout the movement [23]. In addition, the ability to produce greater levels of eccentric force when squatting may indicate that more cross bridges exist during a CMJ, which is a biomechanically similar exercise. Thus, there may be strong relationships between 1RM-BS and CMJ performance. Our final aim was to determine the validity of the CMJ as an Ecc strength assessment method. Previous studies have reported that CMJ has been established as a measure of neuromuscular fatigue and sport performance [62]. Among the CMJ parameters, the mean value with the best reliability may show the actual state [63,64]. In this study, the test data analysis referred to previous studies, and the results were close to the average of the two test parameters for statistical analysis to ensure data reliability [39,65]. The results show that the CV of the EccPF and EccMF indicators of the CMJ are less than 10%, which can be deemed acceptable [46]. Moir et al. (2018) reported that CMJs may provide a means of assessing the Ecc force characteristics [24]. 

The bias estimate of EccPF agreement between the CMJ and 1RM-BS was 1.31, and the bias estimate of EccMF agreement between the CMJ and 1RM-BS was 1.67. The bias estimate variance ratio is 1.31–1.67; if the ratio of variances is >1, this results in a moderate-large correlation in the Bland-Altman plot [66]. When there is a linear trend in the Bland–Altman plot the variability of the differences between methods increases as the magnitude of the measurement increases. In this case, the ratio of measurements on the y-axis of the Bland–Altman plot is used while retaining the same x-axis as the standard Bland–Altman plot [67]. This transformation is equivalent to the logarithmic transformation of data and is the recommended procedure when the assumption of homoscedasticity is violated [68,69].

### Limitations of this Study

This is a cross-sectional study whose results are only inferred from an observational study that analyzes data from a population, or a representative subset, at a specific point in time. The maximal strength likely varied between individuals, and the extent to which strength levels played a role in the reliability of different measures is unclear [19]. However, in this study, EccPF and EccMF reliability was consistent between the 1RM-BS and CMJ. Furthermore, previous studies have shown good day-to-day reliability of the CMJ test [44].

## 5. Conclusions

The CMJ can be established as a dynamic Ecc strength assessment method, and future research can further verify the ECC force value with different equipment measurements (i.e., Sportesse custom-built 45° incline leg press machine, Exerbotics squat device) [23,70]. The combination of a CMJ and force plate measurement is a possible alternative assessment to detect lower extremity dynamic Ecc strength in terms of simplified time, equipment, and manpower. The Ecc peak and mean force values of the CMJ had good reliability and validity and were significantly correlated with the 1RM-BS. In the future, practitioners of related sports science in practical applications can use the CMJ as an Ecc dynamic muscle strength test as a reference for training plans.

## Figures and Tables

**Table 1:** Mean ± standard deviations (SD), coefficients of variation (CV), Kolmogorov-Smirnov normally distributed CMJ and 1RM-BS measures, with 95% confidence intervals (CI), intraclass correlation coefficients (ICC) for the CMJ.

Variable	Mean ± SD	CV %	ICC (95% CI)	Kolmogorov-Smirnov *p* Value
CMJ				
JH (cm)	38.92 ± 5.94	3.81	0.947	0.200 *
EccPF (N)	1980.80 ± 394.34	3.14	0.976	0.100
EccMF (N)	1476.29 ± 331.76	3.31	0.979	0.136
1RM-BS				
Load (kg)	159.54 ± 28.12	9.04	-	0.086
EccPF(N)	2560.99 ± 403.72	9.75	-	0.200 *
EccMF (N)	2393.98 ± 374.66	9.15	-	0.200 *

* Significance lower limit and all parameters *p* > 0.05 are normally distributed.

**Table 2:** Correlations between the CMJ and 1RM-BS measurements for the EccPF and EccMF.

Variable	CMJ-EccPF	CMJ-EccMF	BS-load	BS-EccPF	BS-EccMF
CMJ-JH	0.267	0.232	0.321	0.325	0.274
CMJ-EccPF		0.939 **	0.596 **	0.694 **	0.720 **
CMJ-EccMF			0.474 **	0.578 **	0.616 **
BS-load				0.955 **	0.951 **
BS-EccPF					0.986 **

** indicates significant correlation (*p* < 0.01).

## Data Availability

The data presented in this study are available on request from the corresponding author.
